# Activity of imipenem/relebactam and comparators against KPC-producing *Klebsiella pneumoniae* and imipenem-resistant *Pseudomonas aeruginosa*

**DOI:** 10.1007/s10096-023-04735-1

**Published:** 2023-12-29

**Authors:** Mercedes Delgado-Valverde, Inés Portillo-Calderón, Manuel Alcalde-Rico, M. Carmen Conejo, Carmen Hidalgo, Carlos del Toro Esperón, Álvaro Pascual

**Affiliations:** 1 UGC Enfermedades Infecciosas y Microbiología Clínica, Instituto de Biomedicina de Sevilla (IBIS), Hospital Universitario Virgen Macarena/CSIC/Universidad de Sevilla, Sevilla, Spain; 2 Centro de Investigación Biomédica en Red en Enfermedades Infecciosas (CIBERINFEC), Sevilla, Spain; 3 Instituto de Biomedicina de Sevilla, Hospital Universitario Virgen Macarena, CSIC, Universidad de Sevilla, Sevilla, Spain; 4 Departamento de Microbiología, Universidad de Sevilla, Sevilla, Spain

**Keywords:** Imipenem/relebactam, *P. aeruginosa*, *K. pneumoniae*, Carbapenem-resistant

## Abstract

**Purpose:**

Relebactam is a novel β-lactamase inhibitor, which, when combined with imipenem/cilastatin, is active against both class A and class C β-lactamases. To evaluate in vitro antimicrobial activity of imipenem/relebactam against a collection of recent clinical isolates of carbapenem-non-susceptible *P. aeruginosa* and *K. pneumoniae* ST258 and ST512 KPC producers belonging to different lineages from hospitals in Southern Spain.

**Methods:**

Six hundred and seventy-eight isolates were tested: 265 *K. pneumoniae* (230 ST512/KPC-3 and 35 ST258/KPC-3) and 413 carbapenem-non-susceptible *P. aeruginosa*. Imipenem, piperacillin/tazobactam, ceftazidime, cefepime, aztreonam, ceftolozane/tazobactam, meropenem, amikacin, ciprofloxacin, colistin, and ceftazidime/avibactam were used as comparators against *P. aeruginosa*. Against *K. pneumoniae* ceftazidime, cefepime, aztreonam, and ceftolozane/tazobactam were not tested, and tigecycline was studied instead. MICs were determined in duplicate by broth microdilution according to EUCAST guidelines.

**Results:**

Imipenem/relebactam displayed potent in vitro activity against both sequence types of KPC-3-producing *K. pneumoniae*. MIC_50_ and MIC_90_ values were 0.25 mg/L and 1 mg/L, respectively, with percent of susceptible isolates >97%. Only three *K. pneumoniae* ST512/KPC-3 isolates and one ST258/KPC-3 were resistant to imipenem/relebactam. Relebactam sensitized 98.5% of *K. pneumoniae* isolates resistant to imipenem. The activity of imipenem/relebactam against *P. aeruginosa* was moderate (susceptibility rate: 62.7%). Analysis of the acquired and mutational resistome of isolates with high levels of resistance to imipenem/relebactam has not shown a clear association between them.

**Conclusion:**

Imipenem/relebactam showed excellent activity against *K. pneumoniae* KPC-3. The activity of imipenem/relebactam against imipenem-resistant *P. aeruginosa* was moderate.

**Supplementary Information:**

The online version contains supplementary material available at 10.1007/s10096-023-04735-1.

## Introduction

Carbapenem-resistant Gram-negative bacilli (CRGNB) are a major public health problem, recently described by WHO as a global crisis [1]. Since nosocomial and healthcare-associated infections caused by CRGNB organisms significantly increase morbidity and mortality, length of hospital stay, and medical costs [2], the development of new antimicrobials or new combinations of β-lactam-β-lactamase inhibitors active against these pathogens has become a priority [3, 4]. *Pseudomonas aeruginosa* and *Klebsiella pneumoniae* are common pathogens in hospitals, have a high propensity to develop antibiotic resistance, and also a high capacity for dissemination in the nosocomial environment [5–7]. *P. aeruginosa* carbapenem resistance is driven by different resistance mechanisms, which often act synergistically. The most common mechanisms of imipenem resistance in this microorganism include repression or inactivation of the carbapenem porin OprD coupled with hyperexpression of the chromosomal cephalosporinase AmpC and/or overexpression of efflux pumps, as well as carbapenemase production [5, 8, 9]. In *K. pneumoniae*, the most frequent resistance mechanism to carbapenem is carbapenemase production, mainly of classes A (KPC), B (MBL), and D (OXA-48-like) β-lactamases [10]. In 2017, the WHO published a priority list of pathogens for which the development of new antibiotics was urgently required. Carbapenem-resistant Enterobacterales are included on this list, as well as carbapenem-resistant *P. aeruginosa* and carbapenem-resistant *Acinetobacter baumannii* [11]*.*

Relebactam is a novel diazabicyclooctane β-lactamase inhibitor, which in combination with imipenem/cilastatin is active against class A and class C β-lactamase-producing microorganisms [12, 13]. Imipenem/relebactam was approved by the EMA and FDA in 2020 for the treatment of complicated urinary tract infections, complicated intra-abdominal infections, and hospital-acquired and ventilator-associated bacterial pneumonia in adult patients with limited or no alternative therapeutic options [14, 15].

The purpose of this study was to provide data on the comparative in vitro antimicrobial activity of imipenem/relebactam against a collection of recent clinical isolates of carbapenem-non-susceptible *P. aeruginosa* and *K. pneumoniae* ST258 and ST512 KPC producers belonging to different lineages from hospitals in Southern Spain.

## Methods

### Bacterial strain

Isolates of *K. pneumoniae* (*n* = 265, 230 ST512/KPC-3 and 35 ST258/KPC-3) and *P. aeruginosa* (*n* = 399) tested in this study (*n* = 664) were selected from a well-characterized collection held in the Reference Laboratory of the Andalusian program for the surveillance and control of healthcare-associated infections and antibiotic stewardship (PIRASOA Program), based in the Hospital Universitario Virgen Macarena, Seville, Spain [16, 17]. The isolate source was 77.9% clinical (61.1% *K. pneumoniae*, 88.5% *P. aeruginosa*), 14.9% colonization (24.9% *K. pneumoniae*, 8.5% *P. aeruginosa*), 1% environmental (2.6% *K. pneumoniae*, 0% *P. aeruginosa*), and 6.2% non-specified (11.3% *K. pneumoniae*, 3% *P. aeruginosa*). Two isolates of *K. pneumoniae* ST512 were KPC-31 producers. The selected isolates came from 18 hospitals located in the eight provinces of Andalusia. Thirty-three of the isolates (2 *P. aeruginosa* and 31 *K. pneumoniae*) selected were from 2014, 68 (6 *P. aeruginosa* and 62 *K. pneumoniae*) from 2015, 63 (6 *P. aeruginosa* and 57 *K. pneumoniae*) from 2016, 234 (187 *P. aeruginosa* and 47 *K. pneumoniae*) from 2017, 208 (175 *P. aeruginosa* and 33 *K. pneumoniae*) from 2018, 26 (23 *P. aeruginosa* and 3 *K. pneumoniae*) from 2019, and 32 *K. pneumoniae* from 2020. The inclusion criteria for isolate selection were KPC-3 production in *K. pneumoniae* and imipenem resistance in *P. aeruginosa* non-MBL-producers.

### Bacterial identification, molecular epidemiology, and genomic characterization

Identification of the isolates was confirmed in the reference laboratory by MALDI-TOF MS (MALDI-TOF Biotyper 3.1; Microflex Bruker, Madrid, Spain).

PFGE analysis of XbaI (Enterobacterales)- and SpeI (*P. aeruginosa*)-digested DNA was used to determine the degree of genetic relatedness between isolates. Isolates differing by one or more bands in PFGE assays were assigned to different pulsotypes. A dendrogram was created with Bionumerics 8.0 software (BioMérieux), using the Dice coefficient with optimization set at 1% and position tolerance at 1.2% (data not shown).

In-house Miseq sequencing (Illumina, San Diego, CA, USA) was performed on one isolate of each *K. pneumoniae* pulsetype and in resistant imipenem/relebactam *P. aeruginosa* with MIC >4 mg/L*.* Libraries were prepared with the Nextera XT DNA library preparation kit (Illumina) and sequencing with a reagent cartridge, V3 600 cycles (Illumina). CLC Genomic Workbench software (Qiagen, Netherlands) was used for de novo assembly of Illumina reads. Genomes were analyzed in the Center for Genomic Epidemiology resistance and MLST databases from https://www.genomicepidemiology.org/. All pulsotypes assigned to the same MLST were considered to belong to the same clone. Whenever possible, isolates from the same clone with different pulsotypes were selected.

The total antimicrobial resistance gene content of the *K. pneumoniae* sequenced was analyzed in silico using ResFinder v4.1 (https://cge.food.dtu.dk/services/ResFinder/) and the Comprehensive Antibiotic Resistance Database (CARD) (https://card.mcmaster.ca/). *K. pneumoniae* ATCC10031 was used as a reference to compare porin, PBPs, and efflux bomb aminoacid sequences. In addition, one susceptible isolate was selected for each MIC value for the analysis of mutations related to ß-lactam resistance.

For *P. aeruginosa* genome analysis, raw reads were trimmed with Trimmomatic v0.39 excluding those reads exhibiting a Phred quality score <30, and a subsequent analysis of raw read quality was determined by FASTQC v0.11.9 (https://www.bioinformatics.babraham.ac.uk/projects/fastqc/) and MultiQC v1.10.1. [18]. The genomes were de novo assembled with SPAdes v3.13.0 [19], and the quality of the assemblies was evaluated with QUAST v5.0.2 [20]. The identification of antibiotic resistance genes was performed by AMRFinderPlus [21]. The non-synonymous polymorphisms of genes previously described in *P. aeruginosa* as part of the ß-lactam mutational resistome [22] were identified by calling SNP with Snippy v4.6.0 software (https://github.com/tseemann/snippy), mapping the trimmed raw reads of each bacterial isolate with respect the PAO1 reference genome (NC_002516.2).

### Data availability

The genomes were published in the NCBI database under accession no. PRJNA1048341 (*K. pneumoniae*) and PRJNA1048411 (*P. aeruginosa*).

### Antimicrobial susceptibility testing

Susceptibility testing was performed in duplicate by broth microdilution assay, according to international standard ISO 20776-1.[23] Broth microdilution panels for *P. aeruginosa* included the following antimicrobial agents in doubling dilution concentration ranges (mg/L): imipenem/relebactam (0.03/4–64/4), imipenem (0.03–64), piperacillin/tazobactam (0.06/4–64/4), ceftazidime (0.03–32), cefepime (0.25-32), aztreonam (0.5–64), ceftolozane/tazobactam (0.125/4–16/4), meropenem (0.03–64), amikacin (0.5–32), ciprofloxacin (0.06–2), colistin (0.125–8), and ceftazidime/avibactam (0.015/4–16/4). For *K. pneumoniae,* the activities of piperacillin/tazobactam, ceftazidime, cefepime, aztreonam, and ceftolozane/tazobactam were not tested, and tigecycline activity (concentration range 0.015–1 mg/L) was studied instead. Discrepancies between both replicates were verified using the same method. *Escherichia coli* ATCC 25922 and *P. aeruginosa* ATCC 27853 were used as control strains on each day of testing, checking that all MIC values were within the specified EUCAST ranges [24]. EUCAST interpretive criteria were used to interpret MIC values of all antimicrobials tested [25].

## Results

### Antimicrobial activity against KPC-producing *K. pneumoniae*

**Fig. 1:**
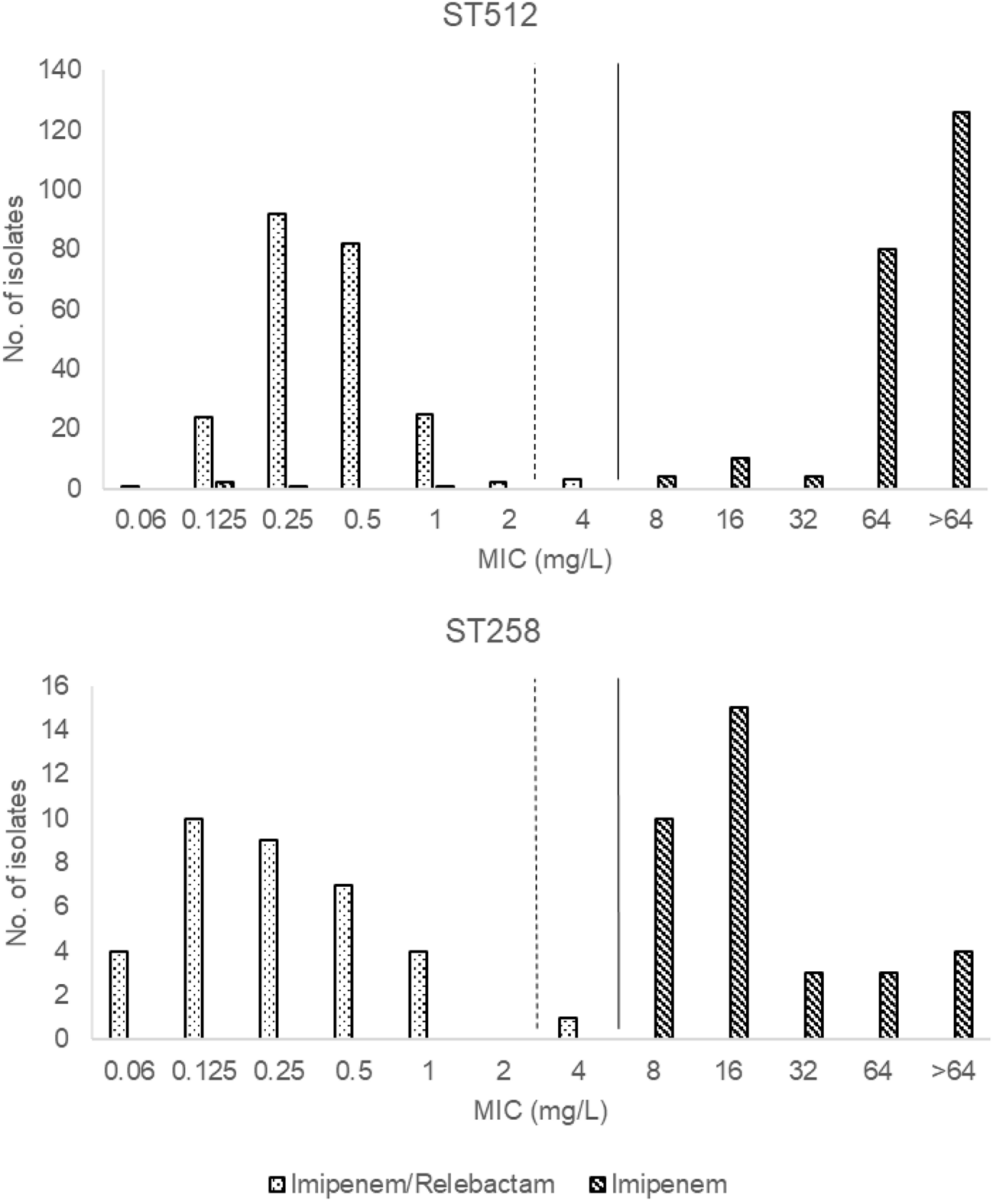
Distribution of imipenem and imipenem/relebactam MICs against *K. pneumoniae* KPC-3 isolates by clone. Vertical dashed line represents the EUCAST resistance breakpoint for imipenem/relebactam (>2/4 mg/L for Enterobacterales) and vertical continuous line represents the EUCAST resistance breakpoints for imipenem (>4 mg/L for Enterobacterales)

Figure 1 shows the MIC distributions of imipenem alone or in combination with relebactam for all *K. pneumoniae* included in the study. The susceptibility rate to imipenem/relebactam was 98.5% (three *K. pneumoniae* ST512/KPC-3 producers and one *K. pneumoniae* ST258/KPC-3 were resistant). With respect to imipenem, only 1.5% of isolates were susceptible. Overall, relebactam sensitized 98.5% of imipenem-resistant isolates (256/260), improving the activity of imipenem by 4 to 8 two-fold dilutions. The MIC range, MIC_50_, MIC_90_ values and percentages of susceptibility and resistance for all isolates are shown in Table 1.

**Table 1:** MIC range, MIC_50_, and MIC_90_ of the tested antibiotics against *K. pneumoniae* KPC-3 by clone and resistance rates

Species/clone/antibiotic	MIC (mg/L)			Resistance (%)		
	MIC range	MIC_50_	MIC_90_	*S*	*I*^2^	*R*
*K. pneumoniae* (*n* = 264)						
Imipenem/relebactam^1^	0.06–4	0.25	0.5	98.5	NA	1.5
Imipenem	0.125–>64	64	>64	1.5	0	98.5
Meropenem	0.5–>64	>64	>64	1.1	3.0	95.9
Ceftazidime/avibactam^1^	0.5–>16	4	8	97.7	NA	2.3
Amikacin	4–>32	>32	>32	1.9	NA	98.1
Ciprofloxacin	2–>2	>2	>2	0	0	100
Colistin	0.125–>16	16	>16	46.2	NA	53.8
Tigecycline*	1–>2	2	>2	0	NA	100
*K. pneumoniae* ST512 (*n* = 229)						
Imipenem/relebactam^1^	0.06–4	0.25	1	98.7	NA	1.3
Imipenem	0.125–>64	>64	>64	1.7	0	98.3
Meropenem	0.5–>64	>64	>64	1.3	0.9	97.8
Ceftazidime/avibactam^1^	0.5–>16	4	8	97.8	NA	2.2
Amikacin	4–>32	>32	>32	1.7	NA	98.3
Ciprofloxacin	2–>2	>2	>2	0	0	100
Colistin	0.125–>16	16	>16	39.7	NA	60.3
Tigecycline*	1–>2	2	>2	0	NA	100
*K. pneumoniae* ST258 (*n* = 35)						
Imipenem/relebactam^1^	0.06–4	0.25	1	97.1	NA	2.9
Imipenem	0.125–>64	>64	>64	0	0	100
Meropenem	0.5–>64	>64	>64	0	17.1	82.9
Ceftazidime/avibactam^1^	0.5–>16	4	8	97.1	NA	2.9
Amikacin	4–>32	>32	>32	2.9	NA	97.1
Ciprofloxacin	2–>2	>2	>2	0	0	100
Colistin	0.125–>16	16	>16	88.6	NA	11.4
Tigecycline*	1–>2	2	>2	0	NA	100

*EUCAST breakpoints for *E. coli*.

^1^For susceptibility testing purposes, the concentrations of relebactam and avibactam were fixed at 4 mg/L

^2^I—susceptible, increased exposure

Imipenem/relebactam and ceftazidime/avibactam displayed similar activity (98.5% susceptibility, MIC_50_ = 0.25/4 mg/L, MIC_90_ = 0.5/4 mg/L compared to 97.7% susceptibility, MIC_50_ = 4/4 mg/L, MIC_90_ = 8/4 mg/L, respectively). Analysis of MIC_90_ values revealed that imipenem/relebactam was four times more active than ceftazidime/avibactam. All ceftazidime/avibactam-resistant isolates were susceptible to imipenem/relebactam. Two of the five isolates resistant to ceftazidime/avibactam produced KPC-31.

There were no differences in imipenem/relebactam MIC values between ST512 and ST258 isolates (MIC_90_ of 1/4 mg/L for both; *p* = 0.8).

The next most active antimicrobial was colistin (46.2% susceptibility, MIC_50_ = 16 mg/L, MIC_90_ = >16 mg/L).

In general, all antimicrobials tested showed similar percentages of susceptibility regardless of MLST, except for colistin, with ST258 isolates being more susceptible than ST512 isolates (88.6% vs 39.7%; *p* < 0.01). All isolates were also resistant to ciprofloxacin and tigecycline.

Analysis of chromosomal mutations in the porin genes and PBPs showed no differences between isolates susceptible and resistant to imipenem/relebactam (Table 2). All isolates had wild-type OmpK36 and AcrAB-TolC regulator (RamR). Mutations in OmpK35 and OmpK37 were detected in two isolates resistant to imipenem/relebactam, although the same mutations were detected in susceptible isolates.

**Table 2:** ST, carbapenemase, antimicrobial susceptibility testing, and mutational ß-lactam resistance genotype of imipenem/relebactam-resistant and susceptible *K. pneumoniae*

Strain no.*	ST	CP	MIC (mg/L)								ß-lactam resistance genotype†						
			MEM	AK	CIP	COL	TIG	IPM	IMR	CZA	ß-lactalactamase	OmpK35^1^	OmpK36	OmpK37^2^	AcrAB-TolC (RamR)	PBP1^3^	PBP2^4^
2015351	258	KPC-3	>64	>32	>2	0.25	1	>64	4	4	SHV-11	mut	wt	mut	wt	mut	mut
20171161	512	KPC-3	16	16	>2	2	1	8	4	0.5	SHV-11	mut	wt	mut	wt	mut	mut
20190807	512	KPC-3	>64	>32	>2	>16	>2	>64	4	8	SHV-11	wt	wt	mut	wt	mut	mut
20190822	512	KPC-3	>64	>32	>2	>16	2	>64	4	4	SHV-11, TEM-1	wt	wt	mut	wt	mut	mut
2016080	258	KPC-3	8	>32	>2	0.5	1	8	0.06	1	SHV-11	mut	wt	mut	wt	mut	mut
2017964	512	KPC-3	>64	>32	>2	1	2	64	0.125	8	SHV-11	mut	wt	mut	wt	mut	mut
20180393	512	KPC-3	>64	>32	>2	>16	1	64	0.25	2	SHV-11	mut	wt	mut	wt	mut	mut
20180260	512	KPC-3	>64	>32	>2	>16	2	>64	0.5	4	SHV-11	mut	wt	mut	wt	mut	mut
20180214	512	KPC-3	>64	>32	>2	>16	2	>64	1	8	SHV-11	mut	wt	mut	wt	mut	mut
2016252	512	KPC-23	>64	>32	>2	1	2	64	2	8	SHV-11, TEM-1	mut	wt	mut	wt	mut	mut

*MEM* meropenem, *AK* amikacin, *CIP* ciprofloxacin, *COL* colistin, *TIG* tigecycline, *IMP* imipenem, *IMR* imipenem/relebactam, *CZA* ceftazidime/avibactam, *PBP* penicillin binding protein, *mut* mutation detected, *wt* wild type

*One susceptible isolate has been included for each MIC value obtained

†*K. pneumoniae* ATCC10031 was used as reference to compared porin, PBPs, and efflux bomb aminoacid sequences

^1^M2_Y36*del*, G37E, M39W, V40S

^2^N230G, M233Q, M233_T234*ins*HYTH, Q235E, T236R, N238Y, R240K, E244D, N247S, D275T, D275_G276*ins*SSTNGG, V277I

^3^I17V, V296_P297*ins*PAQ, V297P, H657R, S662R

^4^D98G, T348S, G515S

### Antimicrobial activity against imipenem-resistant *P. aeruginosa*

**Fig. 2:**
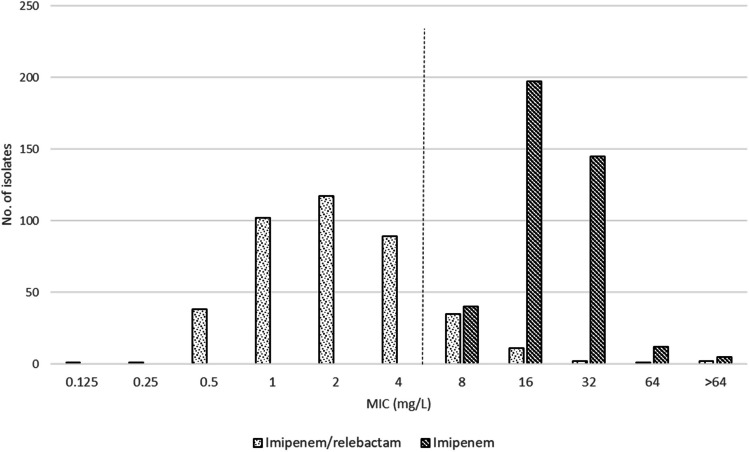
MIC distribution of imipenem and imipenem/relebactam against *P. aeruginosa*. Vertical dashed line represents the EUCAST resistance breakpoint for imipenem/relebactam (>2/4 mg/L for *P. aeruginosa*)

Overall, the new antimicrobials imipenem/relebactam, ceftazidime/avibactam, and ceftolozane/tazobactam showed moderate activity (% susceptibility): imipenem/relebactam (62.7%), ceftazidime/avibactam (73.3%), and ceftolozane/tazobactam (78%). Amikacin was the most active antimicrobial (91.1% susceptibility), followed by colistin (86.7%) (Table 3). The MIC range, MIC_50_, MIC_90_ values, and percentages of susceptibility and resistance for all antimicrobials tested are shown in Table 2. Analysis of MIC_90_ and MIC_50_ values revealed that imipenem/relebactam was two to four-fold more active than imipenem. The MIC distributions of imipenem/relebactam and imipenem are shown in Fig. 2.

**Table 3:** MIC range, MIC_50_, and MIC_90_ of the tested antibiotics against non-carbapenem-susceptible *P. aeruginosa* and resistance rates

Antimicrobial	MIC (mg/L)			Resistance (%)		
	MIC range	MIC_50_	MIC_90_	*S*	*I*^2^	*R*
Imipenem/relebactam^1^	0.125–>64	2	8	64.1	NA	35.9
Imipenem	8–>64	16	32	0	0	100
Piperacillin/tazobactam^1^	1–>32	32	>32	0	39.3	60.7
Ceftazidime	1–>32	16	>32	0	35.6	64.4
Ceftazidime/avibactam^1^	0.5–>32	8	32	76.2	NA	23.8
Ceftolozane/tazobactam^1^	0.25–>16	2	16	80.5	NA	19.5
Cefepime	1–>32	16	>32	0	37.6	62.4
Aztreonam	0.5–>64	32	>64	0	48.9	51.1
Meropenem	1–>64	16	32	4.0	38.1	57.9
Amikacin	0.125–>32	4	16	92.7	NA	7.3
Ciprofloxacin	0.03–>2	2	>2	0	43.1	56.9
Colistin	0.125–>16	1	4	86.0	NA	14.0

^1^For susceptibility testing purposes, the concentrations of relebactam, tazobactam, and avibactam were fixed at 4 mg/L

^2^I—susceptible, increased exposure

**Fig. 3:**
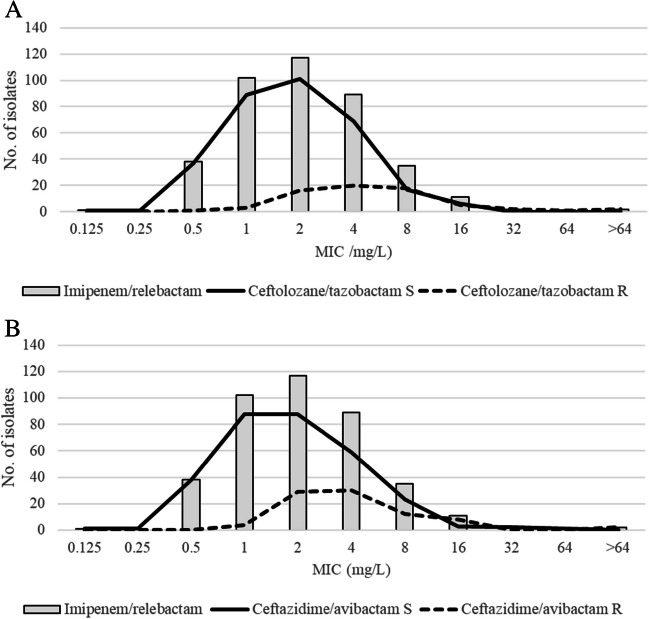
MIC distribution of imipenem/relebactam according to clinical category of ceftolozane/tazobactam (**A**) and ceftazidime/avibactam (**B**) against *P. aeruginosa*. Vertical dashed line represents the EUCAST resistance breakpoint for imipenem/relebactam (>2/4 mg/L for *P. aeruginosa*)

Among the ceftolozane/tazobactam-resistant isolates, 32.6% were susceptible to imipenem/relebactam, while 70.4% of isolates were susceptible to both antimicrobials. Forty-three imipenem/relebactam-susceptible strains were resistant to ceftazidime/avibactam (39.4%). Both antimicrobials were active against 213 isolates (70.1%). In addition, 13.6% of isolates were resistant to ceftazidime/avibactam plus ceftolozane/tazobactam. In this group, the imipenem/relebactam susceptibility rate was 35.7% (20/56 isolates). MIC distribution of imipenem/relebactam according to ceftolozane/tazobactam and ceftazidime/avibactam clinical category is shown in Fig. 3.

Among the colistin-resistant isolates, 51.8% were found to be susceptible to ceftolozane/tazobactam and ceftazidime/avibactam, and up to 58.9% were susceptible to imipenem/relebactam. On the other hand, one-third (32.4%) of the amikacin-resistant isolates were susceptible to relebactam activity.

Regarding WGS results, of the 51 isolates sequenced, only 46 could be analyzed due to technical problems. MLST analysis showed a high variability in our collection. Twenty-six different sequences were detected, with two major ST: ST175 (*n* = 9) and ST274 (*n* = 4). The remaining STs were represented by 3 or less isolates, and one isolate could not be characterized by this method. The 88.8% of isolates from ST175 had an imipenem/relebactam MIC = 8 mg/L, but among isolates with this MIC value, up to 20 different STs were detected, so there does not show a clear association between clone and MIC in isolates with this MIC value.

The analysis of genes involved in ß-lactam resistance is shown in the supplementary table. With respect to chromosomal ß-lactamases analysis: 19 OXA-50 variants were detected among the isolates, with the wild-type variant being the predominant one (*n* = 12), followed by the OXA-488 (*n* = 6) and OXA-486 (*n* = 4) variants, and 18 AmpC variants (PDC-type). The most frequent PDC variants were PDC-1 (*n* = 12) and PDC-3 (*n* = 5). The most frequent polymorphism found in the PDC variants was T105A (71.7%), detected in all PDC alleles except in PDC-1. In general, an association between OXA-50 and PDC variants and ST was detected. The presence of acquired ß-lactamases was detected in only 11 isolates, mostly OXA-10 or OXA-10-like (*n* = 8).

For the mutational analysis of ß-lactam resistance, the polymorphisms present in at least 50% of the *P. aeruginosa* collection were selected, and the results are shown in Table 4. The genes were grouped into 8 functional categories (Table 4 and Supplementary table). Polymorphisms were detected in genes of all categories analyzed. The functional categories and the prevalence of polymorphisms detected were MexXY-OprM and its regulators (30.1%), OprD (18.5%), AmpC and its regulators (13.9%), other PBPs (9.6%), MexEF-OprN and its regulators (8.4%), other ß-lactamases (8.2%), MexAB-OprM and its regulators (7.6%), and LPS modification and RND efflux system regulator (3.9%). In the functional categories AmpC and its regulators, OprD, and other PBPs, none of the polymorphisms was present in more than 90% of the isolates. All isolates presented the mutations K329Q, W358R in MexX of the MexXY-OprM system. Other mutations detected with a high frequency (>95%) were NalC (G71E), MexT (F172I), ParS (H398R), MexY (T543A), ArmZ (L88P), and PIB-1 (I106V) (Table 4).

**Table 4:** Prevalence, function, and variability of mutated genes and polymorphisms detected in more than 50% of imipenem/relebactam-resistant *P. aeruginosa* isolates

Gene†	*V* of mutated genes (%)	Pv of mutated gene (%)	Antibiotic resistance association	Polymorphism	Pv of polymorphisms (%)		
					Total	IMR MIC 8 mg/L	IMR MIC ≥16 mg/L
AmpC and its regulators							
PA0807(*ampDh3*)	1.7	67.4	B, C	A219T	56.5	51.6	66.7
PA4020(*mpl*)	2.0	65.2		M297V	56.5	51.6	66.7
PA4110(*ampC*)	4.7	73.9		T105A	71.7	77.4	60.0
PA4522(*ampD*)	4.0	80.4		G148A	76.1	74.2	80.0
MexAB-OprM regulator							
PA3721(*nalC*)	2.0	**95.7**	B, C, Q	**G71E**	**95.7**	**100**	86.7
				S209R	63.0	67.7	53.3
MexEF-OprN regulators							
PA2491(*mexS*)	2.7	**91.3**	C, Q	**D249N**	**91.3**	**90.3**	**93.3**
PA2492(*mexT*)	1.7	**95.7**		**Q80fs**	89.1	87.1	**93.3**
				**F172I**	**95.7**	**96.8**	**93.3**
LPS modifications and RND efflux system regulation							
PA1798(*parS*)	2.7	**95.7**	A, B, C, P, Q	**H398R**	**95.7**	**96.8**	**93.3**
MexXY-OprM and its regulators							
PA0018(*fmt*)	3.0	**100**	A, B, C, Q	I181V	80.4	77.4	86.7
PA2018(*mexY*)	6.0	**100**		**T543A**	**97.8**	**100**	**93.3**
				Q840E	47.8	41.9	60.0
PA2019(*mexX*)	3.0	**100**		**K329Q**	**100**	**100**	**100**
				L331V	76.1	74.2	80.0
				**W358R**	**100**	**100**	**100**
PA5471(*armZ*)	4.2	**100**		**L88P**	**97.8**	**96.8**	**100**
				D161G	65.2	64.5	66.7
				H182Q	65.2	64.5	66.7
				**V243A**	89.1	**90.3**	86.7
OprD							
PA0958(*oprD*)	12.4	100	C	D43N	52.2	61.3	33.3
				SGS57EGR	58.7	64.5	46.7
				E202Q	63.0	64.5	60.0
				I210A	69.6	74.2	60.0
				E230K	63.0	67.7	53.3
				S240T	65.2	67.7	60.0
				N262T	54.3	54.8	53.3
				A281G	50.0	51.6	46.7
				K296Q	56.5	64.5	40.0
				Q301E	54.3	61.3	40.0
				R310G	45.7	51.6	33.3
				D43N	52.2	61.3	33.3
				SGS57EGR	58.7	64.5	46.7
				E202Q	63.0	64.5	60.0
				I210A	69.6	74.2	60.0
				E230K	63.0	67.7	53.3
Other β-lactamases							
PA5514(*poxB*/OXA-50)	4.2	71.7	B, C	D109E	37.0	29.0	53.3
PA5542(PIB-1)	4.7	97.8		**I106V**	**97.8**	**100**	**93.3**
				S224A	50.0	54.8	40.0
Other penicillin-binding proteins							
PA0869(*pbpG*/PBP6-7)	1.0	69.6	B, C	S250N	56.5	58.1	53.3
PA2272(*pbpC*/PBP3A)	2.7	80.4		A104P	76.1	77.4	73.3
PA4700(*mrcB*/PBP1C)	1.5	71.7		S25G	60.9	64.5	53.3
				L353Q	50.0	51.6	46.7

Prevalence >90% is highlighted in bold

*V* variability (number of polymorphisms found per gene/number of total of polymorphisms), *Pv* prevalence (number of isolates that contains polymorphisms per gene/total isolates), *A* aminoglycosides, *B* non-carbapenem beta-lactams, *C* carbapenems, *Q* quinolones, *P* polymyxins, *IMR* imipenem/relebactam

^†^PAO1 was used as reference genome

## Discussion

One of the results of the increase in MDR-GNB infections worldwide is that the approved antimicrobials provide few treatment options for systemic infections. There is an urgent need for new antimicrobials active against MDR-GNB, as well as sufficient information to facilitate their use in severe infections.

The collection of bacterial strains selected for this study includes KPC-3-producing *K. pneumoniae* isolates from the two most prevalent clones worldwide, as well as the most representative imipenem-resistant non-MBL producer *P. aeruginosa* isolates, both of which cause healthcare-associated infections in Southern Spain (Andalusia has a population of more than 8 million people) and very similar to those causing infections in other neighboring countries.

The results obtained in the current study showed that the in vitro activity of imipenem/relebactam was superior to that of comparators against recent high-risk clone isolates of *K. pneumoniae* KPC-3 producers. Imipenem/relebactam showed potent antimicrobial activity, with MIC_90_ values of ≤1 mg/L against *K. pneumoniae*. MIC_90_ values showed no differences according to the ST tested, as in previous studies [26].

To date, a small number of imipenem/relebactam-resistant *K. pneumoniae* KPC-3 producers have been reported. In our study, 98.5% of KPC-3-producing *K. pneumoniae* were susceptible to imipenem/relebactam. Our results are consistent with those of previous studies. Hernández-García et al. evaluated the in vitro activity of imipenem/relebactam against 14 *K. pneumoniae* KPC-3 producers, all of which were susceptible to imipenem/relebactam [27]. Galani et al. analyzed imipenem/relebactam activity against 314 non-MBL carbapenemase-producing *K. pneumoniae*. Among KPC-producing isolates, 98% were inhibited by this combination, and relebactam effectively restored the in vitro activity of imipenem, with MIC_50_ and MIC_90_ values decreasing from 32/4 to 0.25/4 mg/L, and from >64/4 to 1/4 mg/L, respectively [28]. In a recent study in Spain, 91 KPC-producing isolates were analyzed. The percentage of susceptibility to imipenem was 15.5%, and 100% of the isolates were susceptible to imipenem/relebactam [29].

Ceftazidime/avibactam has been positioned as an alternative for the treatment of infections caused by high-risk clones of KPC-producing *K. pneumoniae*, although the emergence of KPC enzyme variants resistant to this combination has been described, mainly selected after exposure during treatment [30, 31]. Our results agree with those obtained in previous studies, which show that imipenem/relebactam has excellent in vitro activity and clinical efficacy against KPC-producing isolates, even against variants resistant to ceftazidime/avibactam [27, 29, 32]. In our collection, 2.3% of isolates were resistant to ceftazidime/avibactam and all of them were susceptible to imipenem/relebactam. Moreover, MIC_50_/MIC_90_ values were significantly lower than those of ceftazidime/avibactam. Vázquez-Ucha et al. reported similar results, with MIC_50_/MIC_90_ values for imipenem/relebactam and ceftazidime/avibactam of ≤0.25/1 mg/L and 1/8 mg/L, respectively [29].

Several studies have previously shown that reduced porin expression decreases the in vitro activity of imipenem/relebactam. Imipenem/relebactam resistance has been associated with mutations resulting in non-functional OmpK35 and OmpK36 porins in KPC-producing *K. pneumoniae* strains [28, 33, 34]. In our case, we detected four resistant isolates but did not find the mutations associated with resistance to imipenem/relebactam. Since the mutations detected in the porin genes were also present in isolates susceptible to imipenem/relebactam, they cannot explain the resistance to imipenem/relebactam in these four isolates. To our knowledge, there is still very limited data on the clinical efficacy of imipenem/relebactam in patients with severe infections caused by carbapenemase-producing Enterobacterales. The results of the RESTORE-IMI 1 and RESTORE-IMI 2 clinical trials, evaluating the clinical efficacy of imipenem/relebactam for the treatment of infections caused by imipenem-non-susceptible isolates, as well as for treatment of hospital-acquired/ventilator-associated bacterial pneumonia, concluded that imipenem/relebactam was an appropriate treatment option. It should be noted however that the number of carbapenemase-producing isolates was very low [14, 15].

With respect to *P. aeruginosa*, we analyzed a large number of imipenem-resistant non-MBL producer isolates. In our *P. aeruginosa* collection, a susceptibility rate of 62.7% was detected for imipenem/relebactam. Previous studies have reported similar results. The SUPERIOR and STEP studies found a susceptibility rate of 75.7% [35]. Zhang et al. analyzed a collection of 835 non-imipenem-susceptible *P. aeruginosa* isolates from the global SMART surveillance program, and the susceptibility rates to imipenem/relebactam were 64.4%, and the MIC_50_ and MIC_90_ values were 2/4 mg/L and >32/4 mg/L, respectively. Compared with our data, the susceptibility percentages were very similar, but the MIC_90_ value was 2-fold higher [36]. In our study, imipenem/relebactam showed moderate activity against these isolates, as previously described. Young et al. analyzed 3747 isolates of non-imipenem-susceptible *P. aeruginosa*, 714 of which were carbapenemase producers (class A and B). Overall, the MIC value of imipenem/relebactam against 32% of isolates was >4/4 mg/L. This rate was similar to that observed in our study (37.3%), although none of our isolates was a carbapenemase producer [37].

According to the results of our study, approximately one-third of isolates resistant to ceftazidime/avibactam and ceftolozane/tazobactam remain susceptible to imipenem/relebactam. These results are consistent with those previously described in other series [38].

WGS-based analyses of imipenem/relebactam-resistant *P. aeruginosa* isolates show the presence of several acquired OXA-type ß-lactamases, but they generally occur at low prevalence and do not appear to be responsible for the moderate imipenem/relebactam resistance observed among these isolates. Regarding the variants of the chromosomal ß-lactamases found (PDC-type and OXA-50-type), an association is generally observed between the variant detected and the ST rather than with the imipenem/relebactam MIC values obtained, evidencing that other genetic elements should be implicated in resistance to this combined anitibiotic. These data are in agreement with some published studies that showed no significant relationship between acquired OXA-type ß-lactamases or AmpC variants and resistance to imipenem/relebactam [37, 39]. This was also noted by Young et al., who describe that in a collection of 2691 isolates, they found no relationship between imipenem/relebactam MIC and PDC alleles detected in their collection, as well as no association between specific alleles and MIC values [37]. However, the most prevalent AmpC polymorphism found among our imipenem/relebactam-resistant isolates was T105A, previously associated with imipenem increased resistance [40], which was found in all PDC variant detected with the exception of PDC-1. Furthermore, some of the genes widely reported as AmpC regulators were among those genes with high polymorphism prevalence, suggesting that the over-expression of PDC variants with T105A, alone or combined with other polymorphisms, could be relevant for imipenem/relebactam resistance. Our results also showed that the resistance mechanisms with the highest prevalence of polymorphisms among these isolates were detected in the genes related to the MexXY-OprM pumping system and the OprD porin, which is concordant with those described in other studies. Fraile-Ribot et al. reported that resistance to imipenem/relebactam appears to be very low in non-MBL-producing *P. aeruginosa* clinical isolates and isogenic laboratory strains with β-lactam resistance mechanisms that include combinations of OprD inactivation and overexpression of AmpC β-lactamase and/or efflux pumps [38].

In addition, some of the polymorphisms found in our collection had a prevalence of more than 90%. Among ß-lactams resistance–related genes with high prevalence polymorphisms, the MexXY efflux pump system stands out especially, as several of these polymorphisms were observed in both structural components (MexX: K329Q and W358R; MexY: T543A), which could be increasing the affinity of this efflux pump for imipenem or relebactam [39, 41], and in ArmZ regulator (L88P and V243A), which could lead to over-expression of MexXY [42–44]. Moreover, the majority of the isolates in our collection presented the polymorphism (I106V) in chromosomal imipenemase PIB-1, which could be implicated in the increased activity of this enzyme or with a loss of inhibition by relebactam [45]. To confirm this implication, further studies should be necessary. Highly prevalent polymorphisms have also been found in regulators of the MexEF-OprN efflux pump system (MexS: D249N; MexT: Q80fs and F172I), whose relationship with imipenem/relebactam resistance could be more associated with decreased expression of OprD than with over-expression of the MexEF-OprN system itself, as there is no clear evidence of ß-lactam efflux through this RND system [46, 47]. Finally, other polymorphisms were also found in NalC (negative regulator of MexAB-OprM) [48], in ParS (involved in lipopolysaccharide modification and overexpression of some RND efflux pump systems) [49], and in PonA (encoding for PBP1A) [50], all of them with potential involvement in ß-lactam resistance [51], and thus imipenem/relebactam resistance.

Our study has some strengths and limitations. The main strength of this study is that the collection reflects the local epidemiology of a large and specific geographical area. One of the limitations of this study is the absence of WGS data in imipenem/relebactam-susceptible isolates of *P. aeruginosa*, so the prevalence of the polymorphisms among these isolates is unknown. However, taking into account the genomic heterogeneity of the isolate collection analyzed, which includes a high heterogeneity of clones, it is probable that these polymorphisms are directly or indirectly related to imipenem/relebactam resistance in these isolates.

In conclusion, imipenem/relebactam showed excellent activity against *K. pneumoniae* KPC-3 isolates, including those resistant to ceftazidime/avibactam, regardless of sequence type. On the other hand, a moderate number of *P. aeruginosa* isolates were susceptible to imipenem/relebactam and retained activity against some isolates resistant to ceftazidime/avibactam and ceftolozane/tazobactam. Therefore, this combination could be an option to consider in the treatment of infections caused by these microorganisms.

### Supplementary information

ESM 1 (XLSX 24 kb)
